# Rapid *Mycobacterium abscessus* antimicrobial susceptibility testing based on antibiotic treatment response mapping via Raman Microspectroscopy

**DOI:** 10.1186/s12941-023-00644-5

**Published:** 2023-10-30

**Authors:** Weicong Ren, Yuli Mao, Shanshan Li, Bo Gao, Xiaoting Fu, Xiaolu Liu, Pengfei Zhu, Yuanyuan Shang, Yuandong Li, Bo Ma, Luyang Sun, Jian Xu, Yu Pang

**Affiliations:** 1 Department of Bacteriology and Immunology, Beijing Key Laboratory on Drug-Resistant Tuberculosis Research, Beijing Chest Hospital, Capital Medical University/Beijing Tuberculosis & Thoracic Tumor Research Institute, Beijing, 101149 China; 2 Single-Cell Center, Shandong Key Laboratory of Energy Genetics and Shandong Energy Institute, Qingdao Institute of Bioenergy and Bioprocess Technology, CAS Key Laboratory of Biofuels, Chinese Academy of Sciences, Qingdao, Shandong China; 3 University of Chinese Academy of Sciences, Beijing, China; 4 Qingdao Single-Cell Biotech, Co. Ltd, Qingdao, Shandong China

**Keywords:** *Mycobacterium abscessus*, Antimicrobial susceptibility test, D_2_O-probed Raman microspectroscopy, Clarithromycin, Linezolid

## Abstract

**Objectives:**

Antimicrobial susceptibility tests (ASTs) are pivotal tools for detecting and combating infections caused by multidrug-resistant rapidly growing mycobacteria (RGM) but are time-consuming and labor-intensive.

**Design:**

We used a *Mycobacterium abscessus*-based RGM model to develop a rapid (24-h) AST from the beginning of the strain culture, the Clinical Antimicrobials Susceptibility Test Ramanometry for RGM (CAST-R-RGM). The ASTs obtained for 21 clarithromycin (CLA)-treated and 18 linezolid (LZD)-treated RGM isolates.

**Results:**

CAST-R-RGM employs D_2_O-probed Raman microspectroscopy to monitor RGM metabolic activity, while also revealing bacterial antimicrobial drug resistance mechanisms. The results of clarithromycin (CLA)-treated and linezolid (LZD)-treated RGM isolates exhibited 90% and 83% categorical agreement, respectively, with conventional AST results of the same isolates. Furthermore, comparisons of time- and concentration-dependent Raman results between CLA- and LZD-treated RGM strains revealed distinct metabolic profiles after 48-h and 72-h drug treatments, despite similar profiles obtained for both drugs after 24-h treatments.

**Conclusions:**

Ultimately, the rapid, accurate, and low-cost CAST-R-RGM assay offers advantages over conventional culture-based ASTs that warrant its use as a tool for improving patient treatment outcomes and revealing bacterial drug resistance mechanisms.

**Supplementary Information:**

The online version contains supplementary material available at 10.1186/s12941-023-00644-5.

## Introduction

Rapidly growing mycobacteria (RGM) are emerging opportunistic organisms and increasingly important human pathogens [1, 2]. Among them, *Mycobacterium abscessus* (MAB) is the most frequently detected pathogen in individuals suffering from a broad spectrum of diseases, including chronic lung diseases, localized soft tissue infections, and disseminated infections [3, 4]. In fact, pulmonary MAB infection rates are notably high in patients with pre-existing lung conditions, such as bronchiectasis and chronic obstructive pulmonary disease [4, 5]. Meanwhile, patients undergoing various types of immunosuppressive treatments have been reported to have greater rates of disseminated MAB infections than immunocompetent patients [2].

The major threat posed by MAB is its resistance to most classes of antibiotics, including macrolides, aminoglycosides, rifamycins, and β-lactams [4]. The extreme breadth of MAB drug resistance complicates patient treatment management and inevitably leads to poor treatment outcomes, even when newly discovered antimycobacterial agents are administered [4]. Therefore, in order to ensure that MAB-infected patients receive effective antimicrobial therapies, in vitro antimicrobial susceptibility testing (AST) is mandatory prior to treatment initiation, with culture-based phenotypic ASTs currently viewed as gold standard methods for assessing MAB antibiotic susceptibility [6]. However, conventional phenotypic ASTs assess antibiotic effects on bacterial growth and thus require several days to yield drug susceptibility results, especially for bacteria with slow growth rates, such as mycobacteria. To reduce AST turnaround times, researchers have developed ASTs based on microscopic tracking of bacterial cell growth that are more rapid than conventional ASTs [7]. Nonetheless, such methods still require extended periods of time for completion (typically 96 h) in order to generate sufficient numbers of cells to ensure successful automatic or manual MAB detection; such delays lead to delayed initiation of effective anti-MAB antibiotic therapy that can negatively impact patient treatment outcomes.

In recent years, knowledge gained through molecular diagnostics has enhanced our understanding of nucleotide determinants of MAB antibiotic resistance [8–10]. Nevertheless, limited available information on antibiotic resistance mechanisms of drug-resistant clinical MAB isolates has hindered the clinical application of genotypic AST assays for detection of these pathogens [11]. As such, underlying drug resistance genotypes have only been elucidated for MABs with resistance to macrolides, although these efforts have led to the development of a few genotype-based ASTs that can effectively detect MAB mutations conferring macrolide resistance [9, 10]. Despite these successes, the lack of genotypic ASTs that can reliably detect the majority of drug-resistant MAB isolates underscores the urgent need for phenotype-based ASTs that rapidly, accurately, and reliably measure drug susceptibility for use in guiding the formulation of anti-MAB therapeutic regimens.

Raman microspectroscopy, a label-free, non-invasive, and information-rich cellular characterization method, has become an efficient and versatile method for profiling metabolic activities of cell types ranging from microbes [12] to tumor cells [13]. Moreover, methods combining Raman microspectroscopy with stable isotope probing of cells by feeding them heavy water (D_2_O) can enable quantification of antimicrobial drug-induced inhibition of cellular metabolic activity at a single-cell level of resolution [14]. Furthermore, data generated through use of such methods can provide comprehensive information on metabolic responses of bacterial cells to different drugs [15], such that D_2_O-probed Raman microspectroscopy has been successfully used to generate drug resistance profiles for various pathogens [15–19]. However, it is unclear whether such metabolic inhibition-based methods can be effective for assessing mycobacterial drug resistance. Herein, we aim to establish a rapid Raman microspectroscopy-based AST known as Clinical Antimicrobials Susceptibility Test Ramanometry for RGM (CAST-R-RGM) for use in detecting drug-resistant RGM isolates. We then assess the diagnostic accuracy of the CAST-R-RGM assay using a panel of clinical MAB isolates as a mycobacterial model.

## Results

### Overview of CAST-R-RGM workflow

**Fig. 1:**
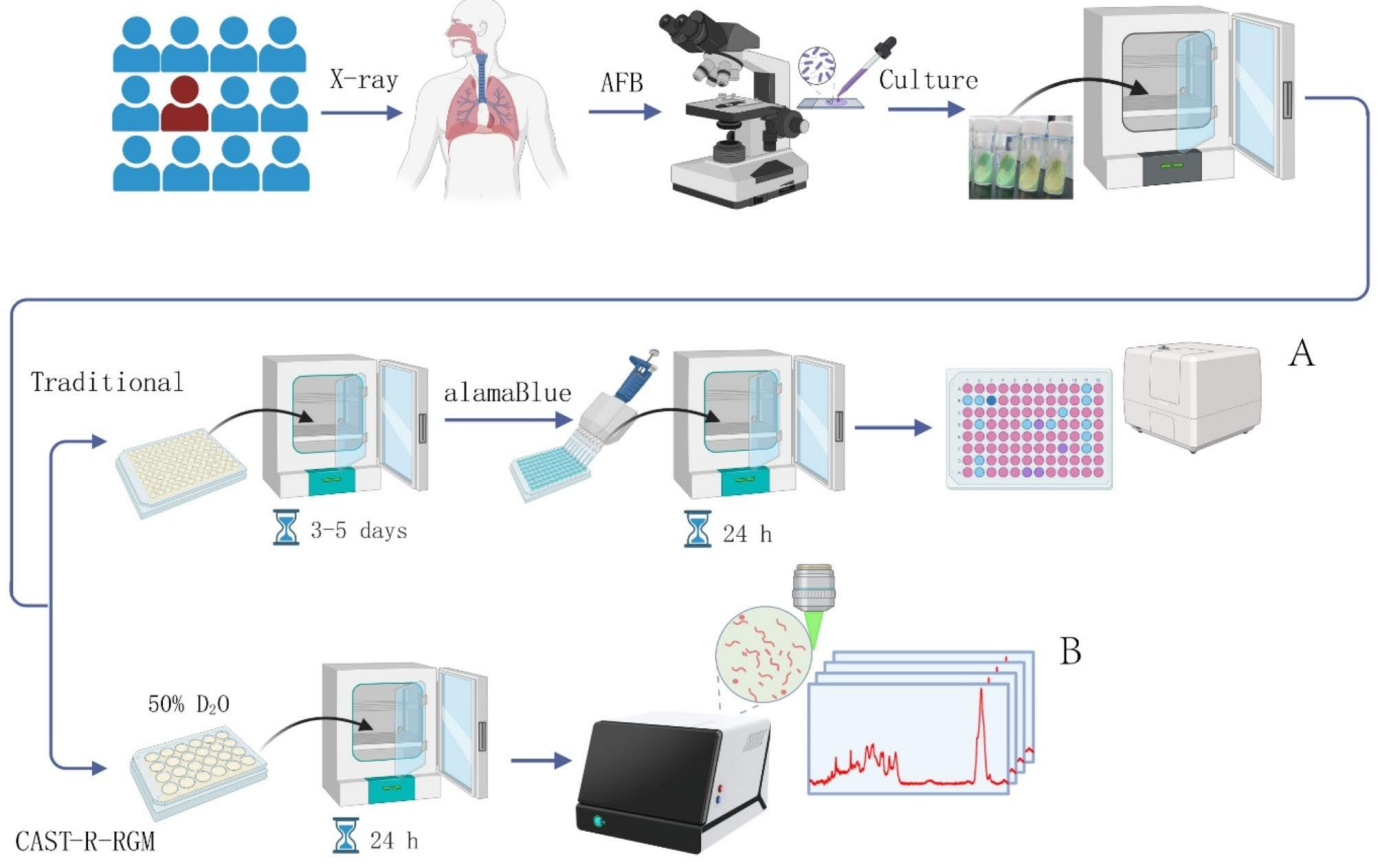
Overview of the CAST-R approach for rapid AST of clinical RGM isolates. The conventional, growth-inhibition-based approach (**A**) and the metabolism-inhibition-based CAST-R-RGM approach (**B**) are both performed for the same samples and then the results were compared side-by-side

Susceptibilities of MAB isolates to antimicrobial agents were determined using the conventional broth dilution-based AST method conducted according to guidelines endorsed by the Clinical and Laboratory Standards Institute (CLSI) [20]. The conventional broth dilution-based method consisted of the following steps: (*i*) clinical isolates were recovered via growth on L-J medium for 7 days; (*ii*) each bacterial suspension was incubated with different concentrations of antimicrobial drugs in the plates; (*iii*) minimum inhibitory concentrations (MICs) results were read after 3–5 days (Fig. 1A).

We had previously developed a method known as D_2_O-probed Raman microspectroscopy (or D_2_O ramanometry) as a cell culture-independent AST [14]. The readout of this AST is based on the relative intensity of C-D Raman band as a measure of antibiotic effects on cellular metabolic activity, which can be expressed quantitatively as the MIC-MA (Minimal Inhibitory Concentration via Metabolic Activity), which was first proposed by our group [14]. Since that time, a Clinical Antimicrobial Susceptibility Test Ramanometry (CAST-R) instrument has been developed that enables automatic acquisition of Raman spectra corresponding to cellular D_2_O intake instead of cellular propagation as a strategy for greatly reducing AST turnaround times for testing of fast-growing pathogens [16].

Based on MIC-MA results obtained through analysis of data collected using the CAST-R system, we developed a rapid RGM AST (CAST-R-RGM) (Fig. 1B). The CAST-R-RGM method consisted of three steps: (*i*) clinical isolates were recovered via growth on L-J medium for 7 days; (*ii*) each clinical isolate was cultured in 7H9 medium containing 50% D_2_O then exposed to a given antibiotic during a 24-h incubation period; (*iii*) >60 Raman spectra of D_2_O-fed, antibiotic-treated cells were acquired and analysed to obtain the final AST results.

### Selection of the optimal D_2_O concentration for conducting CAST-R-RGM

**Fig. 2:**
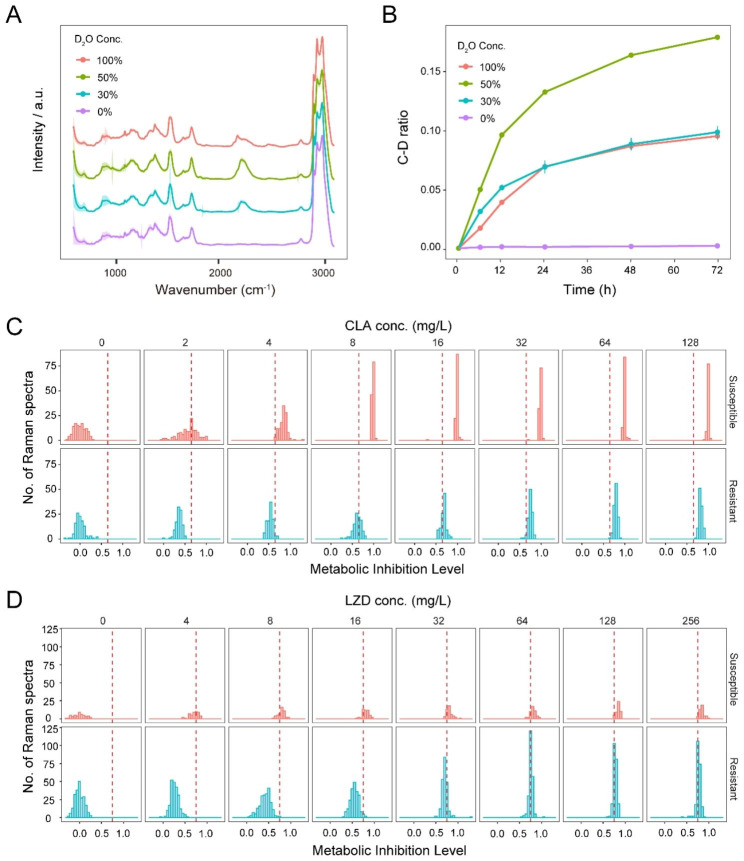
CAST-R-based AST of representative MAB strains for CLA and LZD. (**A**) Change of Raman spectra under an increasing level of D_2_O at 24 h. (**B**) Correlation among C-D ratio, D_2_O concentration and incubation duration for MAB. (**C**) MIL of one CLA-S (MAB ATCC19977) and one CLA-R strain (MAB XA53) under 0-128 mg/L CLA. (**D**) MIL of one LZD-S (MAB ATCC19977) and one LZD-R strain (MAB CS22) under 0-256 mg/L LZD. The red dashed lines are the criteria to distinguish the S and R strains

Since Raman-based ASTs have not yet been reported for any RGM, we initiated the development of such an AST by assessing D_2_O incorporation by MAB ATCC19977 cells during growth in culture medium containing different concentrations of D_2_O. As expected, culturing of live MAB cells in D_2_O-containing medium resulted in the gradual formation of C-D chemical bonds within newly synthesized biomolecules, as reflected by the emergence of C-D Raman band (2040 cm^− 1^-2300 cm^− 1^) in the Raman spectrum. The intensities of these Raman bands increased proportionally with increasing D_2_O exposure duration and dose, as consistent with active bacterial uptake of D_2_O (Fig. 2A). The C-D ratio was proposed and defined as a percentage of the integrated spectral intensity of the C-D band (2040 cm^− 1^-2300 cm^− 1^) to sum of the C-D band and the “C-H band” (2800 cm^− 1^-3100 cm^− 1^), can quantitatively model metabolic activity of the cell. Specifically, C-D ratios obtained for all D_2_O concentrations increased beginning at 6 h of culture and then increased slowly after culture for 24 h. However, incubation of cells with 50% D_2_O concentration produced a broad dynamic range of C-D Raman band, which suggested suitability for comparing its alteration over conditions (Fig. 2B). Therefore, 50% D_2_O was chosen as the default concentration used for the Raman-based MAB AST.

### Raman-based MAB AST developed using CLA and LZD antimicrobial Drugs

To develop a CAST-R-based rapid MAB AST, MAB cells were treated with key drugs used clinically to treat MAB infections (CLA or LZD) as a model [2, 21]. Firstly, to derive a golden reference for interpreting AST results, the “Metabolic Inhibition Level” ($\text{M}\text{I}\text{L}=\frac{{CDR}_{control}-{CDR}_{treated}}{{CDR}_{control}-{CDR}_{0h}}$) was introduced to quantify the extent of cellular metabolic suppression after antimicrobial treatment and normalize against potential variation due to change of strains, starting state of cells, or instruments. Then, MAB organisms that were CLA/LZD-susceptible (strain ATCC19977), CLA-resistant (isolate XA53), and LZD-resistant (isolate CS22) detected by the conventional broth dilution-based AST method were incubated with 50% D_2_O and different drug doses spanned a doubling dilution for 24 h. Specifically, 4 mg/L concentration for CLA and 16 mg/L concentration for LZD were chosen as the breakpoints for Raman-based MAB AST consistent with conventional methods. Moreover, under the breakpoints, alignment of MIL histograms for MAB organisms revealed that maximal separation between sensitive (S) and resistant (R) histograms for CLA concentrations was below 4 mg/L with the cutoff value of MIL designated as “S: ≥0.65, R: <0.65” (Fig. 2C) and for LZD concentrations was below 16 mg/L as “S: ≥0.75, R: <0.75” (Fig. 2D).

### Validation of the Raman-based AST method using a collection of clinical MAB isolates

To assess the performance of the Raman-based AST, both conventional AST and Raman-based AST were conducted in parallel for the same panel of 36 clinical MAB isolates. With conventional culture-based AST, CLA and LZD MIC results served as reference results for use in evaluating Raman-based AST performance (Table S1). Based on conventional AST results, isolates XA99, GZ6, and XA53 were found to be resistant to CLA, while CS22, HN091, HN32, HN67, HN72, XA83, XA93, and XM139 were found to be resistant to LZD.

**Fig. 3:**
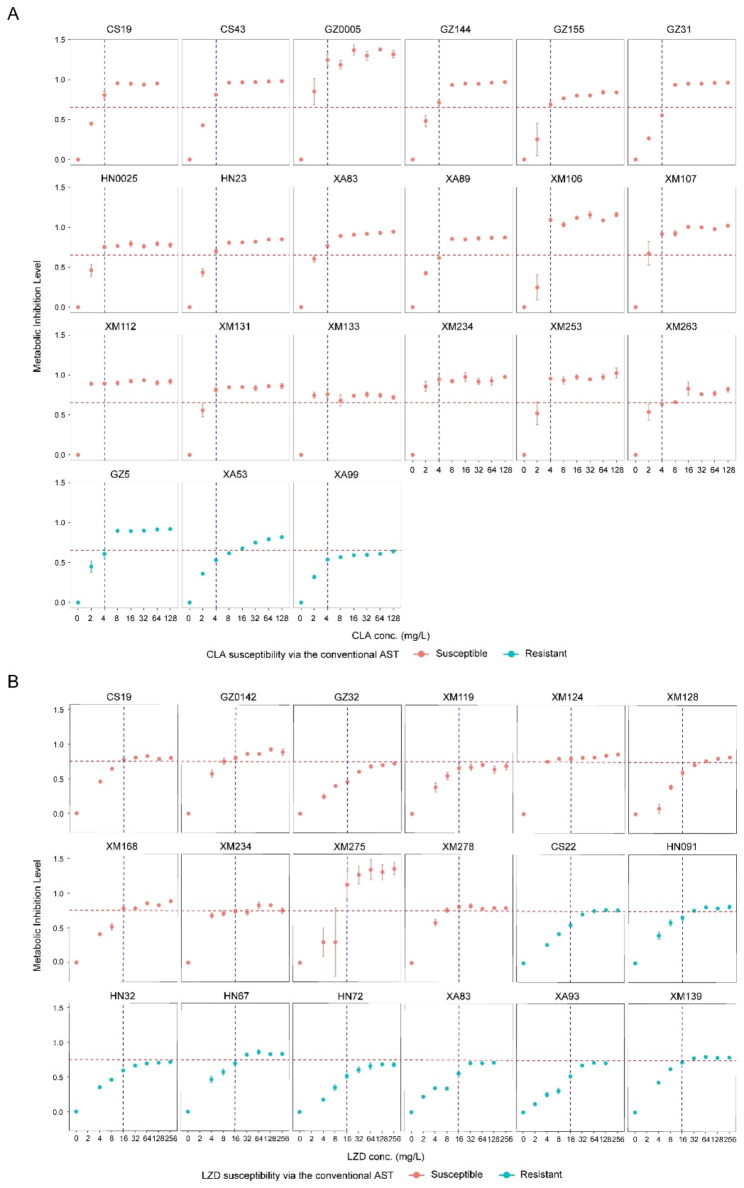
Validation of the CAST-R-based AST for CLA and LZD based on a collection of clinical MAB isolates. The MIL cutoff value was validated with the isolates under 4 mg/L CLA as S: ≥0.65, R: <0.65” (**A**) or 16 mg/L LZD as “S: ≥0.75, R: <0.75” (**B**) after 24 h. The red dashed line is the MIL cutoff value to determine susceptibility

Based on the aforementioned breakpoints, Raman-based AST was performed for the abovementioned clinical MAB isolates. For CLA (red line: MIL of 0.65 as the cutoff value; Fig. 3A), GZ6, XA53, XA89, and XA99 MILs fell below “0.65” and thus were classified as “R”, while MILs of the other isolates exceeded “0.65” and thus were classified as “S.” These results revealed ≥ 90.5% (95% CI 77.9%-100.0%) agreement with results obtained using the conventional AST method (except for GZ31 and XA89, which were falsely classified as “R” according to conventional AST results).

For LZD (red line: MIL of 0.75 as the cutoff value; Fig. 3B), CS22, HN091, HN32, HN67, HN72, XA83, XA93, XM139, GZ32, XM119, and XM128 were classified as “R,” while the other isolates were classified as “S”. These results revealed ≥ 83.3% (95% CI 66.1%-100.0%) agreement between Raman-AST and conventional AST results (except for GZ32, XM119, and XM128 isolates, which were falsely classified as “R” in accordance with results obtained using the conventional AST method).

### RBCS-related insights into drug mechanisms of action

**Fig. 4:**
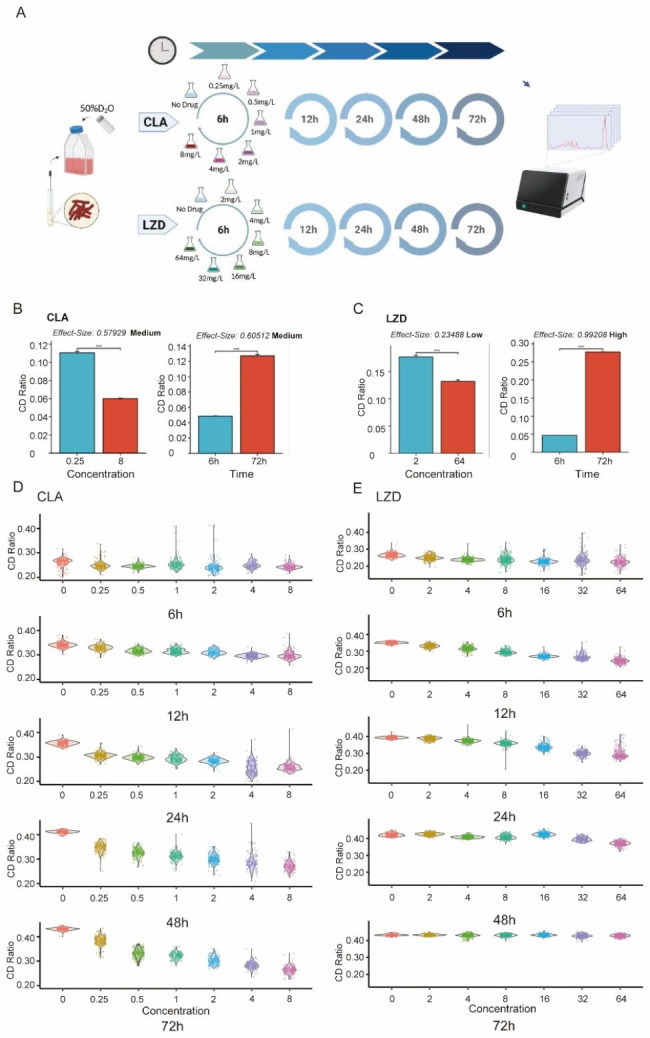
The dynamics of C-D ratio for MAB under various durations of exposure and drug concentrations of CLA and LZD. (**A**) Overview of the experimental design. (**B-C**) Effect size analyses of drug concentration and treatment duration for CLA (**B**) and LZD (**C**). Effect size is calculated by Pearson’s correlation coefficient; P value is calculated by Student’s *t*-test; ***: *p* value < 0.001. (**D-E**) Violin plots showing the dynamics of C-D Ratio under various drug concentrations for treatment with CLA (**D**) and LZD (**E**) for 6 h, 12 h, 24 h, 48 h, and 72 h

To probe CLA and LZD mechanisms of action underlying drug-induced MAB growth inhibition, we tested the standard MAB strain ATCC19977 via Raman-AST using a series of drug-exposure durations and 2-fold dilutions of drug concentrations (Fig. 4A). After identifying all marker Raman bands that were treatment-specific or shared among different treatments, we derived change patterns related to cellular metabolism for the various stress-response programs using the Raman spectra as a proxy.

Based on the abovementioned results, we first asked whether drug exposure duration or drug concentration had a more profound effect on MAB metabolic activity inhibition level. Results of effect size analyses (Materials and Methods) revealed that for CLA, both treatment duration and drug concentration exerted significant effects on the MAB C-D ratio (Fig. 4B), while results obtained for LZD, which were also significant, revealed that treatment duration exerted a more profound effect on C-D ratio than drug concentration (Fig. 4C). Taken together, the abovementioned results revealing different time and dosage effects of the two drugs on cellular metabolic activity suggest that CLA and LZD inhibited MAB growth through different mechanisms.

We then examined MAB C-D ratio changes that occurred in response to increased drug concentrations at various treatment time points. For the CLA group, drug concentration was negatively correlated with the overall C-D ratio, manifesting as a downward trend that became increasingly obvious with increasing treatment duration (Fig. 4D). Meanwhile, similar patterns were observed for the LZD group for the first three time points (6 h, 12 h, and 24 h); interestingly, the C-D ratio became less responsive to LZD after 48-h treatment and was completely irresponsive after 72-h treatment (Fig. 4E).

**Fig. 5:**
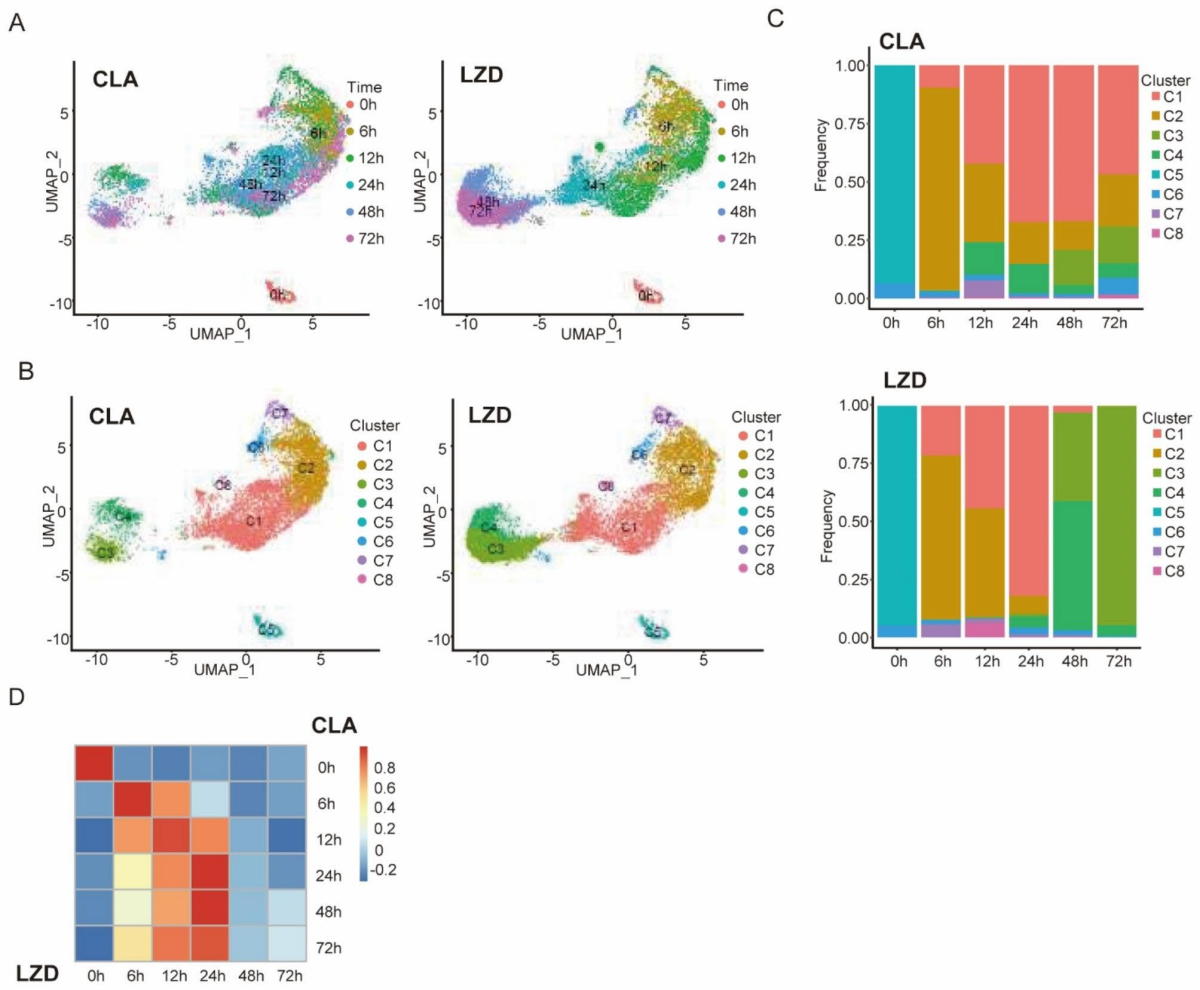
Raman-barcode of cellular-response to stressors (RBCS) of MAB reveals distinct drug mode-of-action in CLA and LZD. (**A**) Dimensionality Reduction plots (UMAP) showing the overall distribution of NTM Raman spectrum data treated with CLA (top) and LZD (bottom). Each dot represents a Raman spectrum. Dots are colored based on the time of treatment. (**B**) UMAP plots as in (**A**) but cells are colored by clusters that were identified by a shared nearest neighbor (SNN) method. (**C**) Stacked barplots showing the percentage composition of clusters at each drug exposure timepoint. (**D**) Heatmaps showing Pearson’s correlation between CLA and LZD at each of the timepoints using data generated in (**C**)

In addition to the D_2_O-associated Raman bands that were used to validate the AST method, thousands of Raman bands were detected in each Raman spectrum that represented specific chemical bonds that could be further identified to yield meaningful biological insights into metabolic responses to drug exposure. In order to fully utilize Raman spectral information and identify key markers related to cellular states induced by drug exposure, RBCS data were obtained for each MAB cell population state then data for different MAB population states were analyzed and compared [15]. Thereafter, global dimensionality reduction was conducted using Uniform Manifold Approximation and Projection (UMAP) analysis for all Raman spectra. Results of this analysis revealed that cells exposed to drug treatments for 0 and 6 h exhibited a strong clustering pattern in the results, forming a distinct and cohesive cluster on their own. By contrast, cells receiving treatments of longer durations (48 and 72 h) exhibited a comparatively greater degree of dispersion (Fig. 5A), thus indicating that long treatment duration had a positive effect on MAB population RBSC diversity. Notably, LZD-treated MAB isolates have a relatively more separated cluster distribution when grouped based on treatment duration, compared to CLA-treated MAB isolates (Fig. 5A). These findings align with our previous observation in Fig. 4B-C, which indicated that the duration of LZD treatment had a greater impact compared to CLA duration, as evidenced by the effect size.

MAB cellular state changes occurring after drug exposures should be continuous processes. To capture transitional cellular state changes occurring after drug exposures, we grouped all MAB isolates into eight clusters based on Raman spectral similarities (Fig. 5B) and then calculated compositions of clusters at each treatment time point (Fig. 5C). Before treatment (0 h), both drug-specific MAB groups (CLA and LZD) were mainly found in Cluster 6, thus confirming that they started from an identical cellular state. Moreover, within the first 24 h of treatment, similar cluster compositions were observed between CLA and LZD (Fig. 5D), thus suggesting a common drug-induced RBCS activation process shared by cells treated with CLA or LZD.

**Fig. 6:**
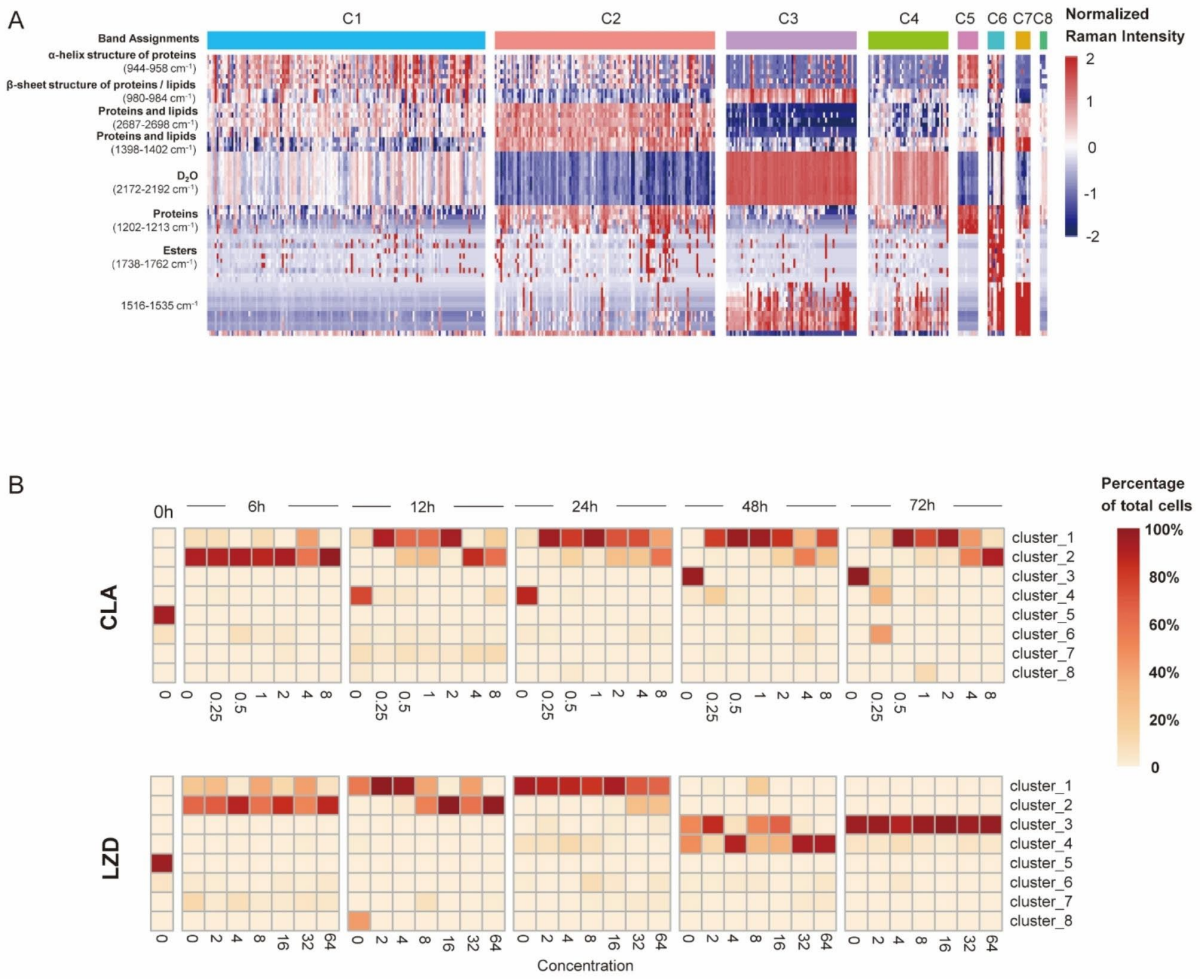
Marker identification and cluster transition with time and drug dosage series. (**A**) Heatmap showing the Raman intensity of the identified markers in each cluster. (**B**) Heatmaps showing the transition of clusters with increased drug concentrations within each treatment timepoint for NTM treated with CLA (top) and LZD (bottom)

We then identified positive markers specific for each cluster (Fig. 6A). Untreated MAB cells were mainly represented by Cluster 6 and produced high-level peak intensities corresponding to ester Raman band (1738–1762 cm^− 1^) and Raman band found in hydroxyproline, tyrosine, phenylalanine, and tryptophan (1202–1213 cm^− 1^). Raman bands of MAB groups treated with CLA and LZD for 6 h were both represented by Cluster 2, due to high-level intensities of protein-, lipid- and D_2_O-associated Raman bands (1202–1213 cm^− 1^, 1398–1402 cm^− 1^, 2687–2698 cm^− 1^). Cluster 1 was the largest group overall and its proportion gradually increased with increasing treatment duration during the first 48 h of CLA treatment and the first 24 h of LZD treatment. As compared with other clusters, this cluster only showed a relatively higher level of α-helix structure-containing proteins (944–958 cm^− 1^). By contrast, Cluster 3 and Cluster 4 were present in groups with long-duration drug exposures, especially those treated with LZD for 48 and 72 h that exhibited high-level intensities of D_2_O Raman band (2172–2192 cm^− 1^) and marker Raman band (1516–1535 cm^− 1^). Compared to Cluster 4, Cluster 3 cells had higher-level intensities of Raman bands found at 980–984 cm^− 1^, which represented structures of protein beta-sheets and some lipids.

Finally, we extended our cluster analyses to include different drug concentrations in order to compare mechanisms of action between the two drugs (Fig. 6B). At 6 h of drug treatment, MAB cells mainly belonged to Cluster 2; at this time point, drug concentration exerted no obvious effects on cluster transition, thus suggesting that 6 h drug treatment was not of sufficient duration for assessment of MAB cell susceptibility to either drug regardless of drug concentration. However, after longer drug exposure durations in CLA, MAB cells gradually transitioned from Cluster 2 to Cluster 1 as drug concentration increased, as reflected by changes in Raman spectrum with higher intensities of Raman bands associated with proteins, lipids and D_2_O (944–958 cm^− 1^, 980–984 cm^− 1^, 2172–2192 cm^− 1^) (Figs. 5C and 6A and B). After 24 h, the number of cells of Cluster 3 were increased which was attributed to long-time accumulation of D_2_O absorption, but Cluster 1 was still dominant. In the case of LZD, during the initial stages of drug treatment, the cells followed a comparable trajectory of cluster transition, moving from Cluster 2 to Cluster 1, accompanied by changes in proteins, lipids, and D_2_O. However, after 24 h, a significant proportion of cells from Cluster 1 underwent a transition to Cluster 3 and Cluster 4, characterized by a decline in protein and lipid (944–958 cm^− 1^, 2687–2698 cm^− 1^) and an increase in D_2_O (2179–2192 cm^− 1^). Eventually, all the cells converted to Cluster 3 at the end of our experiments (72 h). These results are consistent with our previous observations, where MAB cells treated with LZD for 72 h displayed unaltered C-D ratios across different drug concentrations. This suggests that MAB cells exposed to prolonged LZD treatment may undergo additional metabolic remodeling as a mechanism to evade the effects of antimicrobial drugs.

## Discussion

Treatment of MAB infections is challenging, due to drawbacks associated with empiric administration of therapy in the absence of reliable AST results [21]. In this study, we established a rapid, sensitive, and inexpensive diagnostic test, CAST-R-RGM, that could be used to accurately determine CLA and LZD drug resistance for a panel of clinical MAB isolates. This AST is based on the principle that antibiotic treatment dramatically affects bacterial metabolic activity, such that metabolic activity can serve as an accurate proxy of mycobacterial susceptibility to a given antimicrobial drug. In contrast to our previous work employing D_2_O Ramanometry to measure bacterial growth, here the method was used to measure drug effects on cellular metabolic activity. As a consequence, the CAST-R-RGM assay employed the MIC-MA to quantify the minimum drug concentration that could suppress metabolic activity instead of the MIC, which is used as a conventional culture-based AST indicator for quantifying the minimum drug concentration that inhibits cellular growth. In order to explain inconsistencies between results obtained using the two AST approaches, we speculated that the following circumstances may have affected the interpretation of our CAST-R-RGM results: (*i*) for GZ32, the MIC of LZD (16 mg/L) was equal to the critical LZD concentration used to determine LZD susceptibility via the conventional AST; (*ii*) for GZ31, XA89, XM119, and XM128, MILs that fell below 4 mg/L (CLA) or 16 mg/L (LZD) were very close to their corresponding breakpoints of CAST-R-RGM, with results inconsistencies potentially reflecting the fact that the two assays were based on different detection readouts (cellular metabolic activity versus growth).

The CAST-R-RGM method offers several benefits that are not provided by conventional culture-based AST methods. First, for culture-based ASTs drug resistance is defined as the ability of a bacterial population to grow at or above the critical drug concentration threshold. However, the presence of a non-growing bacterial population (referred to as a persister subpopulation) can lead to AST failure [22]. Notably, Lopatkin and colleagues found that antibiotic lethality correlated better with cellular metabolic state than with cellular growth [23]. Our group found that the application of single-cell Raman spectroscopy (SCRS) in the investigation of persisters by treating *Escherichia coli* cells with a lethal dosage of ampicillin for the formation of persisters with different Raman bands and projective zones [24]. Thus, we highlight the promise of cellular metabolism as a more accurate and reliable indicator for use in determining the minimal drug concentration that can kill both fast-growing and non-growing bacteria.

Second, the CAST-R method permitted measurement of metabolic activities of individual cells instead of the average metabolic activity of a potentially heterogeneous cellular population. Thus, use of this method may prevent missed detection of a drug-resistant subpopulation of a larger heterogeneous population, a phenomenon known as heteroresistance, which has been associated with increased risk of poor treatment outcomes for patients afflicted with various bacterial infections. Importantly, results of previous experimental studies have revealed that wild-type drug-susceptible bacteria are generally less fit than drug-resistant forms in the presence of antibacterial drugs and thus can only compete with the drug-resistant subpopulation in the absence of drug pressure [25]. Therefore, the inclusion of a culture step, as is incorporated in conventional culture-based AST methods, may lead to loss of minority drug-resistant subpopulations [26] and failure to detect heteroresistance. By contrast, the D_2_O-Raman assay can be used to determine initial ratios between mutant and WT populations, especially in cases where fitness loss occurs, and thus may outperform culture-based AST methods when used to detect heteroresistance. In view of the fact that heteroresistance is common in mycobacteria [27], results obtained using the CAST-R-RGM method may provide insights into mechanisms underlying drug-induced population heterogeneity with regard to drug resistance to guide the formulation of more effective therapeutic drug regimens.

Finally, the CAST-R-RGM method may serve as a faster and cheaper alternative to conventional ASTs for use in assessing mycobacterial drug resistance. In fact, use of CAST-R-RGM would likely reduce AST turnaround time from that of culture-based ASTs (roughly 3–5 days) to 24 h and thus would enable early treatment initiation and improve treatment compliance. Furthermore, the current CAST-R cost per sample for susceptibility testing for two drugs at four different concentrations is approximately $8 (The cost of the required D_2_O is $2, Raman testing is $5, and other experimental materials is $1), a substantially lower cost than that of conventional AST conducted using the broth dilution method ($15 for mycobacterial susceptibility testing of two drugs). Specially, it has been widely reported for the detection of Mycobacterium tuberculosis via Raman Microspectroscopy [28–30]. Thus, the CAST-R may also apply to *Mycobacterium tuberculosis* which brings greater economic benefits and time savings. Of note, RGM susceptibility testing is not currently conducted in many TB-endemic areas, due to a lack of rapid AST systems in those regions, as well as poor correlations observed between culture-based test results and clinical outcomes. Nevertheless, due to the rise in prevalence of RGM infections, the CAST-R method reported here may serve as a generally applicable quantitative AST, especially in resource-limited settings.

Another interesting finding of this study, which was based on analyses of Raman spectra, revealed dynamic cellular metabolic profile changes associated with cellular responses to drug exposures that depended on drug exposure duration and drug concentration (Figs. 4, 5 and 6). Our results also indicated that drug-challenged MAB cells could be categorized into eight subpopulations based on phenotypic differences in bacterial metabolic remodeling. During the initial 24-h drug exposure phase, bacterial subpopulation compositions exhibited comparable patterns between CLA- and LZD-treated groups, thus suggesting that MAB bacterial cells initiated a non-specific defense mechanism that enabled them to respond to antibiotic-induced stress. Of note, a gradual increase in the proportion of Cluster 1 profiles was observed as a major feature of bacterial subpopulation remodeling, whereby proteins and lipid biosynthesis related markers were generally upregulated upon drug exposure (944–958 cm^− 1^, 980–984 cm^− 1^). This result indicated that bacteria responding to antibiotic exposure may invoke intrinsic multidrug resistance by disrupting cell wall homeostasis [31, 32]. Interestingly, the predominance of Cluster 1 was maintained during 72-h CLA exposure, while LZD exposure led to the continual emergence of bacterial subpopulations with diverse metabolic profiles. These results have important implications, since they suggest that remodeling of cells into cells with a Cluster 1 phenotype may allow them to more effectively escape killing by CLA than by LZD. As a final note, although metabolic variation is usually compared among subpopulations, our single-cell-derived data uncovered the occurrence of a potentially universal mycobacterial drug-induced metabolic remodeling mechanism that enabled transient bacterial survival and supported accumulation of drug resistance-conferring mutations.

We acknowledge several limitations of this study. First, a distinct C-D Raman band served as an indicator of metabolic activity in our analysis, while ignoring the Raman band within the specific D_2_O spectral region that may have provided additional useful information. For example, a Raman spectrum is comprised of discrete Raman bands representing various metabolites (the ramanone), such that a detailed analysis of dynamic mycobacterial responses to antibiotic pressure may reveal correlations between the ramanome and the transcriptome, proteome, or metabolome that could lead to identification of novel targets for use in the rational development of antimycobacterial drugs. Second, since only two representative drugs were used to establish the CAST-R-RGM method, critical drug concentration cutoffs for additional drugs should be determined using the method. Third, despite following the CLSI guidelines for in vitro AST, the bacterial concentrations in the inoculum were not estimated from bacterial counts. Fourth, the CAST-R-RGM method was validated using clinical isolates that were obtained from pure cultures (as confirmed via mass-spectrophotometric analysis) and thus required a long period of time for completion (approximately 10 days). However, CAST-R entails collection of Raman spectra at a single-cell level of resolution for RGM cells that are directly collected from clinical specimens and treated with antimicrobials without the need for isolation of RGMs from pure cultures [33]. Toward this end, experimental devices that efficiently enrich cells from clinical samples and algorithms that distinguish MAB and additional RGM from other commonly encountered clinical microorganisms should be developed.

In conclusion, in this work, MAB served as a model for use in developing a rapid, highly accurate, and affordable CAST-R-RGM assay for detecting MAB isolates with resistance to CLA and LZD. Use of this rapid assay should lead to improved clinical treatment outcomes for patients with MAB infections by supporting earlier treatment initiation with more effective therapeutic regimens. In addition, mining of CAST-R Raman spectra as a rich source of cellular metabolic data will enable researchers to improve AST resolution, assess heterogeneity among drug-exposed MAB cell populations, and enhance our understanding of bacterial metabolic remodeling processes occurring after drug exposure. Further clinical trials are planned to validate the diagnostic performance of this new method using specimens obtained from large cohorts of RGM patients.

## Materials and methods

### MAB isolates, strains, and growth conditions

The *Mycobacterium abscessus* ATCC19977 strain and clinical MAB isolates obtained from several hospitals in different regions of China were stored and maintained at the Department of Bacteriology and Immunology of Beijing Chest Hospital, a hospital affiliated with Capital Medical University. MAB isolates and strain were cultured on Löwenstein–Jensen (L-J) medium at 30 °C. In order to assess the performance of the CAST-R Raman spectroscopy-based AST, two panels of MAB isolates were selected according to in vitro AST results obtained via the conventional broth dilution method and then the AST results were used to evaluate CAST-R diagnostic accuracy. CAST-R diagnostic accuracy in detecting CLA resistance was assessed based on results obtained using the first panel (19 CLA-susceptible and 3 CLA-resistant MAB isolates), while LZD resistance was assessed based on results obtained using the second panel (13 LZD-susceptible and 11 LZD-resistant MAB isolates).

To investigate the effect of D_2_O concentration on bacterial growth, MAB colonies grown on L-J medium were harvested and resuspended in saline solution then glass beads were added to the suspensions followed by vortexing of the tube contents. Next, the turbidity of each resulting suspension was adjusted via dilution to 1 McFarland standard then the suspension was inoculated at a ratio of 1:4 (based on volume) into 10 mL of 7H9 medium (Difco Laboratories, USA) containing 0.05% Tween 80 and either 0%, 30%, 50%, or 100% D_2_O (99.9 atom% D). MAB cultures were then incubated at 30 °C and sampled at 0, 6, 24, and 48 h for Raman spectrum acquisition.

### MIC determinations conducted using a conventional culture-based AST method

The MIC of each antibiotic was defined as the lowest antibiotic concentration for which no bacterial growth was detected. AST was conducted as previously described [34] using a range of CLA concentrations of 0.06 to 16 µg/mL and a range of LZD concentrations of 1 to 32 µg/mL. Briefly, each bacterial suspension (0.5 McFarland standard) was diluted 200-fold in cation-adjusted Mueller-Hinton broth to obtain the initial inoculum. The 100µL of inoculum was added to each well of the plate containing 100 µL/well of serial two-fold dilutions of antimicrobial drugs (CLA or LZD). After plates were incubated for 3–5 days at 30℃, seventy microliters of a freshly prepared 2:5 mixture of Alamar Blue (Bio-Rad, Hercules, CA) reagent and 5% Tween 80 as an indicator of mycobacterial growth was added to each well. The plates were reincubated for one day at 30℃. A pink color indicated growth occurrence, whereas a blue color indicated no detected mycobacterial growth. MIC results were determined based on the lowest drug concentration that prevented a color change from blue to pink.

### MAB CAST-R method incorporating D_2_O-probed Raman spectroscopy

For all CAST-R-RGM-based AST experiments, bacterial cells were suspended in 4 × 7H9 medium. Next, a volume of each cell suspension was added to an equal volume of 4×antimicrobial drug solution in H_2_O and two volumes of a D_2_O stock solution (for a final ratio by volume of 1:1:2) that generated a final culture medium solution containing 1 × 7H9 medium, 1×antimicrobial drug, and 50% D_2_O. After culturing of cell suspensions overnight at 30 °C, samples were collected for Raman spectrum acquisition that was conducted using a CAST-R instrument (Qingdao Single-cell Biotech Ltd., China). All Raman spectra underwent background noise subtraction, baseline correction, and normalization using customized scripts. The C-D ratio, an index used to quantify the extent of substitution of the C-H chemical bond H atom with a D atom as an indicator of cellular metabolic activity, was calculated by dividing the integrated area intensity of the C-D Raman band (2040–2300 cm^− 1^) by the sum of the intensities of the C-D Raman band and C-H Raman band (2800–3100 cm^− 1^) using Ramanome Explorer software (RamEX; Qingdao Single-cell Biotech Ltd, China) [17, 35, 36].

The results were normalized by correcting for species- or strain-specific variations in cellular metabolic responses to drug exposure by introducing the metabolic inhibition level (MIL) into our calculations in place of the ∆C-D ratio, with MIL calculated using the following formula: $\text{M}\text{I}\text{L}=\frac{{CDR}_{control}-{CDR}_{treated}}{{CDR}_{control}-{CDR}_{0h}}$. Mean MIL values obtained for all groups exposed to different doses of an antimicrobial drug ranged from 0 to 1, with a higher MIL value indicating greater inhibition of cellular metabolic activity [16, 33].

### Computation and comparison of Raman Barcodes for Cellular stress (RBCS) response

Each Raman spectrum was preprocessed as described above then low-quality Raman spectra were filtered based on C-H Raman band intensity, the signal-to-noise ratio of the silent region, and principal component analysis (PCA) results using home-made scripts. Local maximum peak regions were identified using the detectPeaks function provided with the MALDIquant R package [37]. Wavenumbers less than 950 cm^− 1^ were filtered out to remove the background signal from the quartz chip during CAST-R spectrum acquisition then the C-D ratio was calculated using the abovementioned method. After quality control was completed, effect size analyses of C-D ratios were conducted for different treatment times and drug concentrations then were assessed for statistical significance as based on Pearson’s correlation coefficient values. P-values were calculated using the Student’s *t*-test.

Raman spectra were merged to generate a large metadata information matrix that was input into Seurat R package [38] for downstream analyses. Three biological replicates of Raman spectra were first integrated as previously reported [38] then the 400 most variable Raman spectral wavenumbers were selected using the FindVariableFeatures function. Thereafter, PCA and UMAP analyses were performed using RunPCA and RunUMAP functions provided with the Seurat package, with the number of principal components (npcs) set to 30 to reduce the number of dimensions. Unsupervised clusters were identified using the FindClusters function using a resolution setting of 0.2. Positive wavenumber markers were identified for each cluster using the “wilcox” test with default parameters. Similarity comparisons between CLA and LZD groups at different time points were performed using Pearson’s correlation analysis.

### Electronic supplementary material

Below is the link to the electronic supplementary material.

Supplementary Material 1

## Data Availability

Not applicable.

## Abbreviations

AST: Antimicrobial Susceptibility Testing
CLA: Clarithromycin
CAST-R: Clinical Antimicrobials Susceptibility Test Ramanometry
CAST-R-RGM: Clinical Antimicrobials Susceptibility Test Ramanometry for RGM
LZD: Linezolid
MAB: *Mycobacterium abscessus*
MIC-MA: Minimal Inhibitory Concentration via Metabolic Activity
MIL: Metabolic Inhibition Level
RBCS: Raman Barcodes of Cellular Stress-response
RGM: Rapidly Growing Mycobacteria
