# Supplementary material for: Rapid Mycobacterium abscessus antimicrobial susceptibility testing based on antibiotic treatment response mapping via Raman Microspectroscopy
Source: Ann Clin Microbiol Antimicrob. 2023 Oct 30;22:94. doi: 10.1186/s12941-023-00644-5 (PMC10617219; doi:10.1186/s12941-023-00644-5)
Supplement: Supplementary file 1 — Supplementary Material 1 [file 12941_2023_644_MOESM1_ESM.docx]

**Supplementary Tables and Figures**

**Table S1. The MICs of 30 clinical isolates via combinations of two kinds of antimicrobials** **by the conventional AST method.**

| **Clinical isolates** | **Antimicrobial susceptibility (MIC (μg/ml))** | |
| --- | --- | --- |
|  | Clarithromycin | Linezolid |
| CS19 | <0.06 | 16 |
| CS43 | <0.06 | -- |
| GZ0005 | <0.06 | -- |
| GZ144 | <0.06 | -- |
| GZ155 | <0.06 | -- |
| GZ31 | <0.06 | -- |
| HN0025 | <0.06 | -- |
| HN23 | <0.06 | -- |
| XA83 | <0.06 | -- |
| XA89 | <0.06 | -- |
| XM106 | 0.12 | -- |
| XM107 | 0.12 | -- |
| XM112 | <0.06 | -- |
| XM131 | <0.06 | -- |
| XM133 | 0.12 | -- |
| XM234 | <0.06 | 4 |
| XM253 | <0.06 | -- |
| XM263 | 4 | -- |
| GZ6 | ≥16 | -- |
| XA53 | ≥16 | -- |
| XA99 | ≥16 | -- |
| GZ0142 | -- | 16 |
| GZ32 | -- | 16 |
| XM119 | -- | 4 |
| XM124 | -- | 4 |
| XM128 | -- | 4 |
| XM168 | -- | 8 |
| XM275 | -- | 8 |
| XM278 | -- | 4 |
| CS22 | -- | ≥32 |
| HN091 | -- | ≥32 |
| HN32 | -- | 32 |
| HN67 | -- | 32 |
| HN72 | -- | 32 |
| XA83 | -- | ≥32 |
| XA93 | -- | ≥32 |
| XM139 | -- | 32 |
